# Development and validation of a claims-based measure as an indicator for disease status in patients with multiple sclerosis treated with disease-modifying drugs

**DOI:** 10.1186/s12883-017-0887-1

**Published:** 2017-06-05

**Authors:** Michael Munsell, Molly Frean, Joseph Menzin, Amy L. Phillips

**Affiliations:** 1 Boston Health Economics, Inc., 20 Fox Road, Waltham, MA 02451 USA; 2 Health Economics & Outcomes Research, EMD Serono, Inc., One Technology Place, Rockland, MA 02370 USA

**Keywords:** Multiple sclerosis, Disease status measure, Retrospective database, Validation, Costs

## Abstract

**Background:**

Administrative healthcare claims data provide a mechanism for assessing and monitoring multiple sclerosis (MS) disease status across large, clinically representative “real-world” populations. The estimation of MS disease status using administrative claims can be a challenge, however, due to a lack of detailed clinical information. Retrospective claims analyses in MS have traditionally used rates of MS relapses to approximate disease status. Healthcare costs may be alternate, broader claims-based indicators of disease activity because costs reflect multiple facets of care of patients with MS, and there is a strong correlation between quality of life of patients with MS and costs of the disease. This study developed, tested, and validated a healthcare cost-based measure to serve as an indicator of overall disease status in patients with MS treated with disease-modifying drugs (DMDs) utilizing administrative claims.

**Methods:**

Using IMS Health Real World Data Adjudicated Claims – US data (January 2006–June 2013), a negative binomial regression predicted annual all-cause medical costs. Coefficients reaching statistical significance (*p* < 0.05) and increasing costs by ≥5% were selected for inclusion into an MS-specific severity score (scale of 0 to 100). Components of the score included rehabilitation services, altered mental state, pain, disability, stiffness, balance disorder, urinary incontinence, numbness, malaise/fatigue, and infections. Coefficient weights represented each predictor’s contribution. The predictive model was derived using 50% of a random sample and tested/validated using the remaining 50%.

**Results:**

Average overall predicted annual total medical cost was $11,134 (development sample, *n* = 11,384, vs. $10,528 actual) and $11,303 (validation sample, *n* = 11,385, vs. $10,620 actual). The model had consistent bias (approximately +$600 or +6% of actual costs) for both samples. In the validation sample, mean MS disease status scores were 0.24, 8.95, and 21.77 for low, medium, and high tertiles, respectively. Mean costs were most accurately predicted among less severe patients ($5243 predicted vs. $5233 actual cost for lowest tertile).

**Conclusion:**

The algorithm developed in this study provides an initial step to helping understand and potentially predict cost changes for a commercially insured MS population.

**Electronic supplementary material:**

The online version of this article (doi:10.1186/s12883-017-0887-1) contains supplementary material, which is available to authorized users.

## Background

Multiple sclerosis (MS) is an inflammatory-mediated, chronic neurodegenerative disease characterized by a range of symptoms including fatigue, impaired motor skills, blurred vision, bladder and bowel dysfunction, and cognitive impairment [1, 2]. The disease has a highly variable prognosis causing early severe disabilities in some patients, but leaving others ambulatory and functional for many years [3, 4]. Comorbidities are also highly prevalent in the MS population, and comorbid disease is recognized as a critical issue in MS given the breadth of adverse impacts with which it is associated [5]. The identification of patients with varying levels of overall disease status is important to help select patient populations most likely to benefit from interventions and to assess the value and effectiveness of treatments [6].

Administrative healthcare claims data provide a mechanism for assessing and monitoring MS disease status in patients with MS across large, clinically representative “real-world” populations [7–9]. Retrospective claims analyses in MS have traditionally used the rates of MS relapses (defined as MS-related hospitalizations, emergency room [ER] visits, or outpatient visits with pharmacy claims for a corticosteroid) as proxy measures for MS disease status [10–19]. Relapses alone, however, do not appropriately capture changes in disease progression and impairment [20]. For instance, as patients with MS progress over time, the number of relapses appears to decrease, despite worsening health status [20]. The estimation of MS disease status using administrative claims can be a challenge, however, due to a lack of detailed clinical information. Retrospective claims analyses in MS have traditionally used rates of MS relapses to approximate disease status. Furthermore, traditional medical models of impairment and disability in MS provide only an incomplete summary because they omit the consideration of comorbidities, secondary conditions, and health behaviors, which may influence the quality of life and disease burden of patients with MS along with biologic variables [21, 22].

Indicators of disease status that incorporate multiple facets of MS may permit assessment of the health status of the patient at a wider level [13, 23–26]. Healthcare costs may be alternate, broader claims-based indicators of disease activity because costs reflect multiple facets of care of patients with MS, and there is a strong correlation between quality of life of patients with MS and costs of the disease [27, 28]. This study utilized an administrative claims dataset to develop, test, and validate a healthcare costs-based measure to serve as an indicator of disease status in patients with MS treated with disease-modifying drugs (DMDs).

## Methods

### Data source and patient population

This retrospective database study used IMS Health Real World Data (RWD) Adjudicated Claims – US data from January 1, 2006 to June 30, 2013. The IMS Health RWD Adjudicated Claims – US database includes complete, adjudicated insurance data, including complete inventory of a patient’s prescriptions, inpatient hospital, and outpatient medical claims. The database consists primarily of patients with commercial health insurance and can thus under-represent the patients with government-paid health insurance (Medicaid or Medicare) relative to patients with private commercial insurance. The database includes ~150 million patients with a medical benefit, and a subset of 95 million patients with both medical and pharmacy benefits.

Patients were aged 18–64 years, had at least one MS diagnosis claim (International Classification of Diseases, Ninth Revision, Clinical Modification [ICD-9-CM] code: 340.xx), and at least one claim for a DMD between January 1, 2007 and June 30, 2012. The date of the first DMD prescription was designated as the index date. Patients were included if they had continuous eligibility 12 months pre- and post-index. Patients were excluded if they had any indication of pregnancy.

### Model development

The data were divided into two samples: an original development sample and a validation/test sample (each comprised 50% of the total patient population). Patients were randomized using the “surveyselect” procedure in Statistical Analysis Software (SAS). A “seed” was set for the randomization process; therefore, the same patients were assigned to the same group every time the analysis was run (i.e., results were therefore replicable). The goal was to create claims-based measures of disease status, one specific to MS and the other focused on general health, using various comorbidity and MS symptom-related codes, as well as additional variables (i.e., sex, age, census region, adherence, newly treated). The general health measure, which uses common Clinical Classification System (CCS) and Charlson criteria, is well-suited for the population as it provides a more complete view of the MS patient’s health, and general health concerns can greatly increase costs among the MS population. MS and a general health measures were included in order to ensure the range of inputs that could affect the algorithm were captured. Costs were used as a proxy for disease status. The analysis evaluated healthcare costs using constant US dollars (i.e., costs were adjusted for inflation using the medical care component of the Consumer Price Index).

Multiple steps were implemented to achieve this goal. First, a negative binomial regression analysis was performed to predict all-cause total direct medical costs (excluding DMD costs) during the follow-up period. Regression covariates included 16 MS-related condition indicators (identified by diagnosis codes), 18 CCS codes, and 17 Charlson-Deyo comorbidities. Condition coefficients that reached both statistical (*p* ≤ 0.05) and economic (MS condition indicators, ≥5% increase in costs; general condition indicators [CCS and Charlson-Deyo], ≥20% increase in costs) significance were included in two normalized scores: an MS score and a general health score (Table 1). Additional file 1: Table S1 and Additional file 2: Table S2 provide the regression results and details of the MS and general score composition that were obtained in the development of the measures.

**Table 1:** MS and general health score components and points

MS score		General health score	
Parameter	Points	Parameter	Points
Rehabilitation	25.597	Myocardial infarction (Charlson)	20.823
Altered mental state	15.802	Metastatic solid tumor (Charlson)	19.027
Pain	12.946	Any primary malignancy (Charlson)	12.172
Disability	10.000	Drug/device complication (CCS)	10.453
Balance disorder	9.211	Diabetes with chronic complications (Charlson)	8.937
Stiffness	8.651	Hematologic (CCS)	6.251
Urinary incontinence	6.366	Gastrointestinal disease (CCS)	5.891
Numbness	4.779	Psychiatric (CCS)	5.785
Malaise and fatigue	4.271	Rheumatologic (Charlson)	5.424
Infections	2.377	Genitourinary (CCS)	5.238

*CCS* Clinical Classification System, *Charlson* Charlson-Deyo comorbidities, *MS* multiple sclerosis

Secondly, coefficients included in each score were re-weighted on a scale from 0 to 100, such that weights represented each predictor’s relative contribution to disease status, as measured by costs (Table 1). Finally, the original negative binomial regression model was re-evaluated to predict costs as a function of the two scores, together with remaining covariates not included in the models for the MS or general health score. The fit of the two models was compared using Bayesian information criteria (BIC). The analysis demonstrated that the model including the MS and general health score was superior to the full regression model at predicting total direct medical costs (score model BIC: 227,340, full model: 227,508; a BIC difference > 10 demonstrates very strong evidence) [29].

### Model validation and testing

As the scores are intended to represent disease status as measured by costs, the MS score, the general health score, and the model combining them were tested and validated in the remaining 50% of the patient population (validation/test population) by assessing the relationship between the two scores and both the predicted and actual costs.

Patients were divided into separate MS and general health score tertiles (i.e., low, medium, and high disease status based on MS or general health score), and predicted and actual costs were summarized for each tertile. Tertiles were generated by ranking all patients according to their disease status score and then dividing the total population into three equal groups, with group cut-offs defined by score ranking. Patients with tied values were grouped into the same tertile. Tertiles were selected for ease of interpretation and because they effectively presented changes in MS/general disease status scores, as the distribution of patients across tertiles was relatively even. An increase in the proportion of patients with a given condition could be seen in each tertile.

All-cause total direct healthcare cost measures were summarized for each tertile using mean, standard deviation (SD), median, interquartile range, and minimum/maximum. Statistical testing was employed to test the significance of difference in predicted versus actual costs between score groups. Differences between actual and predicted costs were assessed using Wilcoxon–Mann–Whitney tests for both the MS and general health score tertiles. Separate analyses were completed for both MS and general health score tertiles.

Additional validity testing was employed using general measures of model error. The bias and absolute prediction error of the model were calculated for both the original model sample and the remaining 50% validation sample. This exercise was conducted to ensure that model error was consistent when using different patient populations. The equations below were used in the bias mean absolute prediction error (MAPE) analyses. These types of prediction accuracy measures have been used in previous health economic studies (e.g., Austin 2003 compared the accuracy of different regression models used to predict coronary artery bypass graft surgery medical costs) [30].

$$ \mathrm{Bias}=\left(\mathrm{mean}\ \mathrm{of}\ \mathrm{individual}\ \mathrm{predictions}\right)-\left(\mathrm{mean}\ \mathrm{of}\ \mathrm{actual}\ \mathrm{observations}\right). $$

= $$ \frac{1}{n}\sum_k{\hat{Y}}_k-\frac{1}{n}\sum_k{Y}_k $$

MAPE = $$ \frac{1}{n}\sum_k\left|{\widehat{Y}}_k-{Y}_k\right| $$

### Exploration of MS/general health score composition

The proportion of patients with each condition used to calculate the MS and general health score was evaluated for each MS/general health score tertile. This analysis was conducted to determine how disease status factors change as MS/general health score increases.

## Results

A total of 11,384 patients (50%) were included in the original development population, and 11,385 patients (50%) were included in the validation/test population.

A breakdown of the number of patients included in each MS and general health score tertile, as well as overall MS/general health scores for each tertile, are shown in Tables 2 and 3.

**Table 2:** MS score and MS score component frequency within the validation/test population

	MS disease status group		
	Low tertile	Medium tertile	High tertile
Patients, *n*	3902	3801	3682
MS score			
Mean (SD)	0.24 (0.71)	8.95 (2.46)	21.77 (9.23)
Median (IQR)	0.00 (0.00–0.00)	10.00 (9.05–10.00)	19.21 (14.78–23.99)
Minimum	0	4.27	12.92
Maximum	2.38	12.38	91.35
MS score components, *n* (%)			
Rehabilitation	0	0	135 (3.7)
Altered mental state	0	0	133 (3.6)
Pain	0	0	370 (10.0)
Disability	0	2409 (63.4)	3369 (91.5)
Stiffness	0	4 (0.1)	115 (3.1)
Balance disorder	0	362 (9.5)	1823 (49.5)
Urinary incontinence	0	126 (3.3)	425 (11.5)
Numbness	0	317 (8.3)	1223 (33.2)
Malaise and fatigue	0	686 (18.0)	1892 (51.4)
Infections	388 (9.9)	550 (14.5)	731 (19.9)

*IQR* interquartile range, *MS* multiple sclerosis, *SD* standard deviation

**Table 3:** General health score and general health score component frequency within the validation/test population

	General disease status group		
	Low tertile	Medium tertile	High tertile
Patients, *n*	4400	3426	3559
General health score			
Mean (SD)	0.00 (0.00)	5.72 (0.31)	16.07 (7.22)
Median (IQR)	0.00 (0.00–0.00)	5.78 (5.42–5.89)	12.14 (11.13–17.46)
Minimum	0	5.24	8.94
Maximum	0	6.25	64.82
General health score components, *n* (%)			
Myocardial infarction	0	0	47 (1.3)
Metastatic solid tumor	0	0	45 (1.3)
Any primary malignancy	0	0	363 (10.2)
Drug/device complication	0	0	658 (18.5)
Diabetes with chronic complications	0	0	159 (4.5)
Hematologic	0	329 (9.6)	1038 (29.2)
Gastrointestinal disease	0	1033 (30.2)	2301 (64.7)
Psychiatric	0	1190 (34.7)	2090 (58.7)
Rheumatologic	0	31 (0.9)	125 (3.5)
Genitourinary	0	843 (24.6)	1876 (52.7)

*IQR* interquartile range, *MS* multiple sclerosis, *SD* standard deviation

**Fig. 1:**
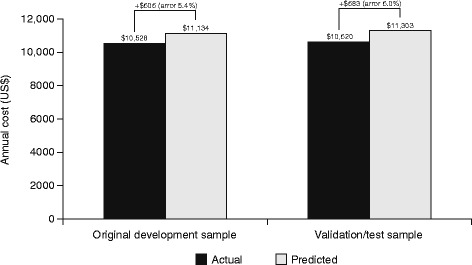
Mean predicted vs. actual annual all-cause total medical costs for original development and validation/test samples*. *All costs adjusted to 2015 US dollars using the medical care component of the Consumer Price Index

Mean MS scores were 0.24, 8.95, and 21.77 in the low, medium, and high MS disease status tertiles, respectively. Patients in the low tertile had a median score of 0 and maximum score of 2.38, corresponding to the presence of an infection. Patients who experienced the three condition indicators associated with the highest scores – rehabilitation services, altered mental state, and pain – were all categorized in the highest tertile. Mean general health scores were 0.00, 5.72, and 16.07 for the low, medium, and high tertiles, respectively. No patients in the lowest tertile had any general health score condition indicators, and patients with any of the five condition indicators associated with the greatest score were all grouped in the highest tertile. Average annual predicted and actual costs in the overall population (i.e., not stratified by disease status tertile) for each group are shown in Fig. 1.

Bias was similar in both models, with predicted costs being approximately 6% higher than actual costs. The mean (SD) absolute prediction error was consistent across both populations: $7274 (15,306) in the original development population and $7387 (17,670) in the validation/test population.

Average annual predicted and actual costs within the validation/test population for the MS score and the general health score disease status tertiles are shown in Table 4.

**Table 4:** Predicted vs. actual costs in the validation/test population, by MS or general health score disease status tertiles^a^

	Disease status tertile		
	Low	Medium	High
MS score			
*n*	3902	3801	3682
Predicted costs, $			
Mean (SD)	5047 (2867)	8583 (5397)	20,743 (34,041)
Median (IQR)	4269 (3393–5712)	7193 (5359–10,042)	12,941 (8926–21,055)
Minimum	2149	2415	3642
Maximum	55,398	82,177	1,046,113
Actual costs, $			
Mean (SD)	5143 (9204)	8923 (12,770)	18,176 (27,936)
Median (IQR)	2688 (1065–6052)	5344 (2617–10,210)	10,215 (5212–19,976)
Minimum	0	0	0
Maximum	241,470	293,690	499,099
General health, score			
*n*	4400	3426	3559
Predicted costs, $			
Mean (SD)	5243 (2630)	8202 (4413)	21,781 (34,500)
Median (IQR)	4507 (3471–6260)	7175 (5239–10,019)	13,973 (9567–22,598)
Minimum	2149	2638	3552
Maximum	39,259	73,002	1,046,113
Actual costs, $			
Mean (SD)	5233 (7947)	8455 (10,609)	19,363 (29,432)
Median (IQR)	2950 (1187–6195)	5510 (2686–10,350)	10,725 (5429–21,577)
Minimum	0	0	0
Maximum	133,669	135,713	499,099

*IQR* interquartile range, *MS* multiple sclerosis, *SD* standard deviation

^a^All costs adjusted to 2015 US dollars using the medical care component of the Consumer Price Index

**Fig. 2:**
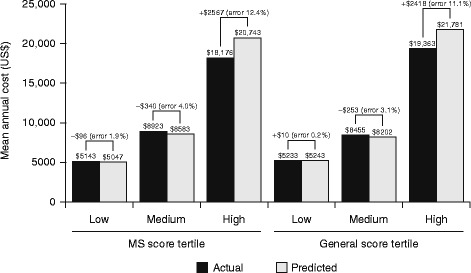
Mean predicted vs. actual annual all-cause total medical costs and general health score tertile. *MS* multiple sclerosis*. *All costs adjusted to 2015 US dollars using the medical care component of the Consumer Price Index

The model predicted average costs most accurately for patients in the lowest disease status tertiles (bias of 0.2–1.9%; Fig. 2).

A bias of 11.1–12.4% ($2567 for MS score, $2418 for general health score) was recorded in the high disease status tertiles (Fig. 2). Differences in predicted vs. actual costs were significantly different across disease status tertiles for both MS and general health scores (*p* < 0.0001 for all).

## Discussion

Overall (i.e., without stratification by disease status tertiles), the average predicted annual direct healthcare cost was $11,134 for the original development sample (vs. $10,528 actual cost) and $11,303 for the validation/test sample (vs. $10,620 actual cost). Therefore, the model had consistent bias (approximately $600, or 6% of actual costs) for both samples. In Austin 2003, other regression-based prediction models had similar degrees of bias [30].

The mean absolute prediction error also remained consistent in both populations (approximately $7000 for the original development and validation/test samples), demonstrating further validity of the model.

The mean absolute prediction error represents the average deviation of each individual predicted value from the actual value, and is therefore more sensitive to outliers than the measure of bias. However, the magnitude of the absolute prediction error in the analysis is comparable with the predictive cost models from Austin 2003, which resulted in a mean absolute error of approximately $6600 for a dataset with mean actual costs of $17,900 [30].

The slightly higher absolute prediction error in this study is most likely due to the magnitude of outliers from evaluating all-cause total direct medical costs vs. event-specific costs (i.e., maximum actual total cost in the analysis was $1,046,113, while maximum actual surgery-specific cost was $166,461 in Austin 2003 [30]).

On average, the model predicted costs most accurately among patients with lower disease status. Specifically, the model under-predicted costs by an average of $96 for the low MS disease status tertile (mean predicted $5047; mean actual $5143; bias 1.9% of actual) and over-predicted by only $10 for the low general health score tertile (mean predicted $5243; mean actual $5233; bias 0.2% of actual). The difference between predicted and actual cost was approximately $2500 for both the highest MS disease status and general health disease status tertiles (bias 11.1–12.4% of actual). Differences in predicted vs. actual costs were significantly different (*p* < 0.0001) between disease status tertiles. The better prediction in the lowest tertile could be explained by the likely lower amounts of variation in this subpopulation compared with the other subpopulations.

The MS score model predicted annual all-cause total medical costs with acceptable estimations. While there does not appear to be a standard threshold for evaluating the accuracy of predictive cost models (i.e., most publications simply evaluate several different models and comment on relative accuracy by comparing model bias), the predictive model appears to be consistent with other published validated models. The bias of models used to predict coronary artery bypass graft surgery costs in the Austin 2003 publication ranged from 3.5% to 19.1% of the actual cost, with the negative binomial regression resulting in a 5.3% bias (vs. 6% bias in this analysis) [30]. An additional analysis that used a logistic regression to predict stroke treatment costs resulted in a 3% bias (range 0–5% of actual cost, depending on the subgroup analyzed), with the paper concluding that the predictive model’s minimal bias directly confirmed its accuracy [31].

There are limitations in this analysis. The ICD-9-CM code for systemic MS does not distinguish between different MS types. Not all indicators of disease status may be captured in all-cause total medical costs as assessed by healthcare claims. Findings could be confounded by cost variations across settings, but relative relationships could be expected to hold. Also, a limited number of patients had very high MS or general health scores (mean disease status scores in the highest tertile did not exceed 25 out of a maximum score of 100 in either condition group). It is, therefore, difficult to evaluate the predictive accuracy of the model at the highest levels of MS disease status that were not present in the database. Finally, there is a possible lack of generalizability of the data given the inherent characteristics of claims databases and sample cohorts. The sample consisted of US patients with commercial claims. US patients with commercial claims are typically younger than 65 years of age, and most likely come from an employed population since they have commercial insurance; therefore, they may have better access to treatment. Also, the magnitude of claims is expected to be different in non-US patients; however, the conditions that are associated with high claims may still be relevant to other countries. Further research in other populations is warranted.

## Conclusions

This analysis demonstrated that claims-based measures that incorporate MS-specific as well as general health components can be used as indicators for disease status in patients with MS treated with DMDs. The performance of the predictive model is consistent with other published validated models. Healthcare decision makers and researchers may use these models to better ascertain the disease status of patients with MS. This may help in identifying patients likely to benefit from intervention and help to assess the value and effectiveness of treatments.

## Additional files

Additional file1: Table S1. Negative binomial regression predicting all-cause total costs (excluding DMD costs). Description of Data: Table S1 provides the regression results that were obtained in the development of the measures. (DOCX 15 kb)

Additional file 2: Table S2. MS and general score composition. Description of Data: Table S2 provides the details of the MS and general score composition that were obtained in the development of the measures. (DOCX 13 kb)

## Abbreviations

*BIC*: Bayesian information criteria
*CCS*: Clinical Classification System
*DMD*: Disease-modifying drug
*ER*: Emergency room,
*ICD-9-CM*: International Classification of Diseases, Ninth Revision, Clinical Modification
*IQR*: Interquartile range
*MAPE*: Mean absolute prediction error
*MS*: Multiple sclerosis.
*SAS*: Statistical Analysis Software.
*SD*: Standard deviation.
