# Supplementary material for: Development and validation of a claims-based measure as an indicator for disease status in patients with multiple sclerosis treated with disease-modifying drugs
Source: BMC Neurol. 2017 Jun 5;17:106. doi: 10.1186/s12883-017-0887-1 (PMC5460356; doi:10.1186/s12883-017-0887-1)
Supplement: Supplementary file 1 — Negative binomial regression predicting all-cause total costs (excluding DMD costs). Description of Data: Table S1 provides the regression results that were obtained in the development of the measures. (DOCX 15 kb) [file 12883_2017_887_MOESM1_ESM.docx]

**Supplementary Table 1** Negative binomial regression predicting all-cause total costs (excluding DMD costs)

| **Parameter** | **Estimate** | **Standard error** | **Wald 95% confidence interval** | | **Wald chi-square** | **Pr > ChiSq** |
| --- | --- | --- | --- | --- | --- | --- |
|  |  |  | Lower | Upper |  |  |
| **intercept** | 7.9148 | 0.1563 | 7.6085 | 8.2211 | 2564.32 | <0.0001 |
| **male** | 0.0222 | 0.0221 | –0.0211 | 0.0656 | 1.01 | 0.3152 |
| **ageAsOfIndex** | –0.0121 | 0.007 | –0.0259 | 0.0017 | 2.97 | 0.0846 |
| **ageSquared** | 0.0002 | 0.0001 | 0 | 0.0003 | 3.56 | 0.0593 |
| **northeast** | 0.2272 | 0.0248 | 0.1786 | 0.2758 | 83.92 | <0.0001 |
| **midwest** | 0.0942 | 0.0245 | 0.0463 | 0.1422 | 14.83 | 0.0001 |
| **west** | 0.1684 | 0.0309 | 0.1079 | 0.2289 | 29.78 | <0.0001 |
| **nonadherent** | 0.2186 | 0.0184 | 0.1825 | 0.2547 | 140.76 | <0.0001 |
| **prevTxPrevDx** | 0.0976 | 0.039 | 0.0212 | 0.174 | 6.27 | 0.0123 |
| **newTxPrevDx** | 0.0292 | 0.0382 | –0.0457 | 0.1042 | 0.58 | 0.4446 |
| **msScore** | 0.0255 | 0.0011 | 0.0233 | 0.0277 | 511.74 | <0.0001 |
| **genScore** | 0.0333 | 0.0015 | 0.0304 | 0.0362 | 506.73 | <0.0001 |
| **mSCIConstipationFoll** | –0.0149 | 0.0451 | –0.1033 | 0.0734 | 0.11 | 0.7403 |
| **mSCIDepressionFollow** | 0.0905 | 0.0255 | 0.0405 | 0.1405 | 12.6 | 0.0004 |
| **mSCIMuscleSpasmFollo** | 0.0001 | 0.0398 | –0.0779 | 0.0781 | 0 | 0.9975 |
| **mSCIMusculoskeletalF** | 0.0021 | 0.1086 | –0.2108 | 0.2149 | 0 | 0.9849 |
| **mSCIOpticNeuritisFol** | 0.0669 | 0.0346 | –0.0009 | 0.1347 | 3.74 | 0.0532 |
| **mSCISpirometryFollow** | 0.1352 | 0.0672 | 0.0035 | 0.2669 | 4.05 | 0.0441 |
| **cCSArthBoneJointFoll** | 0.1994 | 0.1086 | –0.0134 | 0.4122 | 3.37 | 0.0662 |
| **cCSCancerFollowUp** | 0.1543 | 0.0279 | 0.0996 | 0.2089 | 30.62 | <0.0001 |
| **cCSCVSFollowUp** | 0.1579 | 0.0204 | 0.1178 | 0.1979 | 59.65 | <0.0001 |
| **cCSDermatologicFollo** | 0.0343 | 0.0218 | –0.0085 | 0.0772 | 2.47 | 0.1158 |
| **cCSEndocrineFollowUp** | 0.0924 | 0.0233 | 0.0468 | 0.1379 | 15.78 | <0.0001 |
| **cCSEyeEarFollowUp** | 0.0606 | 0.0196 | 0.0223 | 0.099 | 9.6 | 0.0019 |
| **cCSGYNDiseaseFollowU** | 0.1076 | 0.0202 | 0.068 | 0.1471 | 28.41 | <0.0001 |
| **cCSInfectiousDisease** | 0.0753 | 0.0196 | 0.037 | 0.1137 | 14.81 | 0.0001 |
| **cCSInjuriesFollowUp** | 0.1385 | 0.0205 | 0.0982 | 0.1787 | 45.46 | <0.0001 |
| **cCSIntraAbdomOrganFo** | 0.0977 | 0.036 | 0.0271 | 0.1683 | 7.35 | 0.0067 |
| **cCSMetabolicNutritFo** | 0.09 | 0.0288 | 0.0334 | 0.1465 | 9.73 | 0.0018 |
| **cCSPulmonaryFollowUp** | 0.1635 | 0.0236 | 0.1171 | 0.2098 | 47.82 | <0.0001 |
| **cCSVenousFollowUp** | 0.2331 | 0.0375 | 0.1596 | 0.3067 | 38.58 | <0.0001 |
| **charlsonAIDSFollowUp** | 0.2609 | 0.4738 | –0.6677 | 1.1896 | 0.3 | 0.5818 |
| **charlsonCHFFollowUp** | 0.3165 | 0.1073 | 0.1062 | 0.5268 | 8.7 | 0.0032 |
| **charlsonCOPDFollowUp** | 0.04 | 0.0355 | –0.0297 | 0.1096 | 1.26 | 0.2608 |
| **charlsonCVAFollowUp** | 0.1486 | 0.0476 | 0.0554 | 0.2418 | 9.76 | 0.0018 |
| **charlsonDementiaFoll** | 0.0125 | 0.2198 | –0.4182 | 0.4433 | 0 | 0.9545 |
| **charlsonDiabNoCCFoll** | 0.1469 | 0.0398 | 0.0688 | 0.225 | 13.59 | 0.0002 |
| **charlsonHemiParaFoll** | 0.2789 | 0.0654 | 0.1508 | 0.407 | 18.2 | <0.0001 |
| **charlsonMildLiverFol** | 0.1196 | 0.1732 | –0.2199 | 0.4592 | 0.48 | 0.4898 |
| **charlsonModSevLiverF** | –0.7595 | 0.5535 | –1.8443 | 0.3253 | 1.88 | 0.17 |
| **charlsonPepticUlcerF** | 0.1412 | 0.1221 | –0.0981 | 0.3806 | 1.34 | 0.2474 |
| **charlsonPVDFollowUp** | 0.2737 | 0.0995 | 0.0787 | 0.4687 | 7.57 | 0.0059 |
| **charlsonRenalFollowU** | –0.1153 | 0.1056 | –0.3222 | 0.0916 | 1.19 | 0.2749 |

*DMD* disease-modifying drug
