# Supplementary material for: Development and validation of a claims-based measure as an indicator for disease status in patients with multiple sclerosis treated with disease-modifying drugs
Source: BMC Neurol. 2017 Jun 5;17:106. doi: 10.1186/s12883-017-0887-1 (PMC5460356; doi:10.1186/s12883-017-0887-1)
Supplement: Supplementary file 2 — MS and general score composition. Description of Data: Table S2 provides the details of the MS and general score composition that were obtained in the development of the measures. (DOCX 13 kb) [file 12883_2017_887_MOESM2_ESM.docx]

**Supplementary Table 2** MS and general score composition

| **Parameter** | **Points** |
| --- | --- |
| **MS-specific score** | |
| Rehabilitation | 25.597 |
| Altered mental state | 15.802 |
| Pain | 12.946 |
| Disability | 10.000 |
| Stiffness | 8.651 |
| Balance disorder | 9.211 |
| Urinary incontinence | 6.366 |
| Numbness | 4.779 |
| Malaise and fatigue | 4.271 |
| Infections | 2.377 |
| **General score** | |
| Myocardial infarction (Charlson) | 20.823 |
| Metastatic solid tumor (Charlson) | 19.027 |
| Any primary malignancy (Charlson) | 12.172 |
| Drug/device complication (CCS) | 10.453 |
| Diabetes with chronic complications (Charlson) | 8.937 |
| Hematologic (CCS) | 6.251 |
| Gastrointestinal disease (CCS) | 5.891 |
| Psychiatric (CCS) | 5.785 |
| Rheumatologic (Charlson) | 5.424 |
| Genitourinary (CCS) | 5.238 |
| *CCS* Clinical Classifications System, *Charlson* Charlson-Deyo comorbidities, *MS* multiple sclerosis | |
